# Computer-aided diagnosis using embedded ensemble deep learning for multiclass drug-resistant tuberculosis classification

**DOI:** 10.3389/fmed.2023.1122222

**Published:** 2023-06-26

**Authors:** Kanchana Sethanan, Rapeepan Pitakaso, Thanatkij Srichok, Surajet Khonjun, Nantawatana Weerayuth, Chutinun Prasitpuriprecha, Thanawadee Preeprem, Sirima Suvarnakuta Jantama, Sarayut Gonwirat, Prem Enkvetchakul, Chutchai Kaewta, Natthapong Nanthasamroeng

**Affiliations:** ^1^Department of Industrial Engineer, Faculty of Engineering, Research Unit on System Modelling for Industry, Khon Kaen University, Khon Kaen, Thailand; ^2^Department of Industrial Engineer, Faculty of Engineering, Artificial Intelligence Optimization SMART Laboratory, Ubon Ratchathani University, Ubon Ratchathani, Thailand; ^3^Ubon Ratchathani University, Department of Mechanical Engineer, Faculty of Engineering, Ubon Ratchathani, Thailand; ^4^Division of Biopharmacy, Faculty of Pharmaceutical Sciences, Ubon Ratchathani University, Ubon Ratchathani, Thailand; ^5^Ubon Ratchathani University, Division of Biopharmacy, Faculty of Pharmaceutical Sciences, Ubon Ratchathani, Thailand; ^6^Department of Computer Engineering and Automation, Faculty of Engineering, Kalasin University, Kalasin, Thailand; ^7^Department of Information Technology, Faculty of Sciences, Buriram Rajabhat University, Buriram, Thailand; ^8^Department of Computer Science, Faculty of Computer Sciences, Ubon Ratchathani Rajabhat University, Ubon Ratchathani, Thailand; ^9^Department of Engineering Technology, Faculty of Industrial Technology, Ubon Ratchathani Rajabhat University, Ubon Ratchathani, Thailand

**Keywords:** tuberculosis, drug resistant, computer aided diagnosis, ensemble deep learning, artificial multiple intelligence system

## Abstract

**Introduction:**

This study aims to develop a web application, TB-DRD-CXR, for the categorization of tuberculosis (TB) patients into subgroups based on their level of drug resistance. The application utilizes an ensemble deep learning model that classifies TB strains into five subtypes: drug sensitive tuberculosis (DS-TB), drug resistant TB (DR-TB), multidrug-resistant TB (MDR-TB), pre-extensively drug-resistant TB (pre-XDR-TB), and extensively drug-resistant TB (XDR-TB).

**Methods:**

The ensemble deep learning model employed in the TB-DRD-CXR web application incorporates novel fusion techniques, image segmentation, data augmentation, and various learning rate strategies. The performance of the proposed model is compared with state-of-the-art techniques and standard homogeneous CNN architectures documented in the literature.

**Results:**

Computational results indicate that the suggested method outperforms existing methods reported in the literature, providing a 4.0%-33.9% increase in accuracy. Moreover, the proposed model demonstrates superior performance compared to standard CNN models, including DenseNet201, NASNetMobile, EfficientNetB7, EfficientNetV2B3, EfficientNetV2M, and ConvNeXtSmall, with accuracy improvements of 28.8%, 93.4%, 2.99%, 48.0%, 4.4%, and 7.6% respectively.

**Conclusion:**

The TB-DRD-CXR web application was developed and tested with 33 medical staff. The computational results showed a high accuracy rate of 96.7%, time-based efficiency (ET) of 4.16 goals/minutes, and an overall relative efficiency (ORE) of 100%. The system usability scale (SUS) score of the proposed application is 96.7%, indicating user satisfaction and a likelihood of recommending the TB-DRD-CXR application to others based on previous literature.

## 1. Introduction

In several developing countries, the prevalence of tuberculosis (TB) and the growing elderly population have resulted in a shortage of drug susceptibility testing (DST) for the *Mycobacterium tuberculosis* (Mtb) strain, leading to inappropriate treatment and increased drug resistance (1, 2). The World Health Organization (WHO) has categorized drug resistance into five types based on severity: drug-sensitive TB (DS-TB), drug-resistant TB (DR-TB), multidrug-resistant TB (MDR-TB), pre-extensively drug-resistant TB (pre-XDR-TB), and extensively drug-resistant TB (XDR-TB) (3).

Thailand faces a significant public health challenge with drug-resistant TB, with studies reporting high rates of multidrug-resistant TB ranging from 2.9 to 14.7% (4–6). Research in Thailand has primarily focused on understanding drug resistance, including specific mutations in genes associated with resistance (7). Recent studies have identified novel mutations in drug-resistant TB strains across different regions of Thailand (8). Additionally, drug-resistant TB has been observed among Thai children (5). Molecular methods for TB diagnosis and drug susceptibility testing have played a crucial role in identifying genetic mutations associated with drug resistance in Thai TB strains.

In Nigeria, the prevalence of DR-TB is significant among newly diagnosed TB cases and previously treated TB cases (9). However, traditional drug sensitivity testing (DST) is limited due to resource constraints and the need for advanced laboratory infrastructure, leading to delayed results. Early detection and treatment of DR-TB are crucial to prevent complications and improve outcomes. Chest X-ray (CXR) has proven effective in diagnosing and evaluating tuberculosis, and it has been used in TB screening, triage, and diagnosis (10–14). Various studies have explored the use of CXR images in computer-aided diagnosis (CAD) for drug-resistant TB, achieving notable accuracies (15–19).

Existing research has primarily focused on binary classification of DR-TB (20–22), while practical applications require identifying specific drug resistance classes from a single CXR image, highlighting the need for multiclass classification models. Multiclass classification is challenging but attainable, as demonstrated by previous studies (17–19). To enhance model performance, ensemble deep learning, which combines multiple distinct models, could be utilized for classifying drug-resistant TB types, potentially achieving higher accuracy (23). Ensemble deep learning has shown promise in various medical applications, surpassing the performance of single deep learning models (24).

Computer-aided diagnosis (CAD) technologies can aid in automated detection and screening for drug-resistant TB, particularly in areas lacking radiological expertise (25, 26). Lung and mediastinal areas play crucial roles in TB detection, and combining their differences has been shown to improve diagnostic accuracy (27–29). Image segmentation techniques, such as ensemble models and lung segmentation, have been employed to enhance classification outcomes, yielding promising results (30–34).

Previous studies have employed various machine learning and deep learning approaches, achieving accuracies between 66.0 and 94.9% (35–40). However, there is room for improvement, as multiclass classification accuracy may be lower compared to binary classification. Evaluating ensemble deep learning models using key performance indicators such as AUC, F-measure, and accuracy provides insights into their effectiveness (41, 42). Decision fusion strategies and preprocessing techniques influence the performance of ensemble deep learning (43, 44).

In this research we developed multiclass classification models for drug-resistant TB using CXR images. After obtaining classification model, the web application was developed. The application is capable of diagnosing drug-resistant strains from a single CXR sample, with the program’s output recommending an appropriate treatment regimen. The application is referred to as “TB Drug Resistance Diagnosis System-CXR,” or “TB-DRD-CXR” for short.

Nonetheless, this article makes the following contributions:

1. This is the first-time multiclass classification has been used to categorize drug-resistant strains.
2. Initially, heterogeneous ensemble deep learning was utilized to categorize drug-resistant organisms.
3. This is the first web application that can classify drug resistance in “live” mode and provide the user with a recommended regimen based on the detected drug resistance class.
4. Four classification procedures for “live” classification were proposed in order to identify the most promising strategies for classifying drug resistance “live.”

This work aimed to construct the application TB-DRD-CXR, which incorporates a deep learning model, in order to provide physicians with additional information regarding the CXR analysis performed by AI. If the physicians obtain sufficient and more reliable results, they are not required to adhere to the treatment plan or classification. The structure of this study is as follows: Sections 2 and 3 present the materials/methods and computational results, respectively, while Sections 4 and 5 delve into the discussion and conclusion.

## 2. Related articles

A chest X-ray is the basic radiologic evaluation that is performed when tuberculosis (TB) is either suspected or confirmed (10). When used in conjunction with other symptoms and signs, a CXR offers high sensitivity in the diagnosis of tuberculosis (11). Infection with the human immunodeficiency virus (HIV), the degree of immunosuppression prior TB treatment, and a microbiological profile that is drug-sensitive (DS), have all been shown to change CXR outcomes in patients who have tuberculosis (12). Some studies have indicated that DS-TB and DR-TB manifest differently on a CXR in terms of the shape, size, and location of the lesions (12, 13), and CXRs have played a significant role in the screening, triage, and diagnosis of TB (14).

Computer-aided diagnosis (CAD) technologies have been used to reduce human error while extracting information from CXR images (25). These technologies can aid in the automated detection and screening of populations for drug-resistant tuberculosis, particularly in locations devoid of radiological expertise. The primary components of a CAD system are region-of-interest (RoI) segmentation, feature extraction from the RoI, and feature-based classification (26). The lungs and the mediastinal area are extracted from the CXR image. When searching for tuberculosis, specialists must examine these two locations thoroughly. The lungs surround the thoracic mediastinum, which is located at the top of the chest (18). Lung and mediastinal deformation have been associated with the most common TB abnormalities, such as pleural effusions, consolidation, cardiomegaly, fibrosis, infiltration, bronchial dilation, and mediastinal lesions (27). Both Cheng et al. (28) and Cha et al. (29) concluded that combining lung and mediastinal differences can improve the diagnostic accuracy when identifying whether a patient has a different medication response.

As explained in Rahman et al. (30), the ensemble image segmentation model combines two models into a single system to classify image patches and merge them into a pre-segmentation. The first model is a traditional convolutional neural network (CNN). The second model is a tweaked version of the U-Net architecture (31), which first separates the patches and then merges them into a single image. The initial segmentation is created by joining these two pre-segmented images using a binary disjunction operation; the final segmentation is obtained via postprocessing. The postprocessing stages include standard image processing methods such as erosion, dilation, connected component identification, and region-filling algorithms. The computed results highlight the significance of lung segmentation in enhancing classification outcomes. Accordingly, scientists think that a good classification result can be achieved by paring down a CXR image to its essential components (32–34).

In Tulo et al. (16), as the primary contribution to the proposed research, the authors employed image segmentation and machine learning (ML) to classify four categories of drug resistance. The machine learning algorithms that were proposed to distinguish between TB-negative, DS, and DR-TB included K-nearest neighbor (KNN), multilayer perceptron (MLP), support vector machine (SVM), and linear discriminant analysis (LDA) (20, 35). Deep learning approaches utilizing various CNN architectures were used to construct an AI drug resistance categorization model (15). Li et al. (27) transformed CT images to the coronal plane using a pretrained ResNet50 convolutional neural network (CNN). Cheng et al. (28) and Cha et al. (29) employed an ensemble of 3D CNNs and a 3D texture-based graph model, and support vector machines (SVMs), respectively, for the same problem. Govindarajan et al. (36) addressed this classification task by replacing the softmax function of a 3D CNN architecture with an SVM. All entries showed minimal success, resulting in AUCs of roughly 0.60. Previously, numerous studies had classified small cases. In Kukker and Sharma (35), the authors examined DR-TB and DS-TB cases from a single hospital, while Toğaçar et al. (25) presented a large study in which the number of DR-TB cases increased from 183 to 468. Using information from 144 individuals, Ramaniharan et al. (26) determined that the existence of numerous cavities is a strong predictor of DR-TB. Thacker et al. (37) compared 516 instances of DR-TB to 1,030 cases of DS-TB. The AUC of all proposed approaches was restricted to 0.83, which had the potential to increase.

Ensemble deep learning is a new deep learning architecture that combines multiple distinct CNN models to improve the effectiveness of the existing model. Using a hybrid ensemble feature extraction approach, Talukder et al. (38) was able to effectively diagnose lung and colon cancer. This approach combines deep feature extraction and ensemble learning with a high performance, and the mode can detect lung, colon, and lung and colon cancer with accuracies of 99.05, 100, and 99.30 percent, respectively. Using pretrained DenseNet-121, DenseNet-201, ResNet-101v2, and ResNet-50 architectures, Barsha et al. (39) proposed an ensemble model for the evaluation of invasive ductal carcinoma (IDC). The model was inferred from two cohorts of validation. On one validation cohort, the model had overall accuracy values of 69.31, 75.07, 61.85, and 60.50% for patch-level classification and 62.44, 79.14, 76.67, and 71.00% filtering for cancer image datasets. The three-stage ensemble-boosted convolutional neural network model was presented in Kalaivani and Seetharaman (40).

In the initial step of processing CXR datasets, a traditional segmentation model (ResUNet) is employed to enhance the model’s performance. The proposed model has an accuracy of 99.35%, which is superior to the traditional model currently in use (40).

The AUC, F-measure, and accuracy are the three KPIs used to assess the quality of ensemble deep learning employed in this research (41, 42). Accuracy measures the number of positive and negative observations that are correctly categorized. Since the precision score is based on the predicted classes, the F-measure is computed as the harmonic mean of precision and recall, giving each variable equal weight. It enables a model to be evaluated by considering both precision and recall using a single score, which is useful when summarizing the performance of a model or comparing models. The AUC (area under the curve) is a performance metric for classifying issues with different threshold values. The receiver operating characteristic (ROC) curve is a probability curve, whereas the area under the curve (AUC) shows the degree or measure of separability. It indicates how well a model can differentiate between classes. The larger the AUC, the more accurately a model predicts 0 classes as 0 and 1 classes as 1. The quality of ensemble deep learning is dependent on numerous parameters, such as the preprocessing techniques (43), the CNN architecture (44), and the model’s decision fusion strategy (38). Long short-term memory (LSTM)-based classifiers improve the usual majority voting approach by proposing the best–worst weighted voting technique to enhance network generalization performance. A heterogeneous ensemble network comprising convolutional neural networks (CNN) and random forest models was used to learn distinct features from acoustic emission (AE) data and categorize the AE signals into their respective phases, as proposed in (41). The results indicated that the suggested model outperformed the conventional model and classified signals into their allocated phases with greater precision.

## 3. Materials and methods

The creation of the CAD to classify the drug resistance types of tuberculosis patients necessitated three research stages. The subsequent actions were as follows: (1) collecting the dataset from previous research; (2) designing an effective ensemble deep learning approach; and (3) developing the Tuberculosis Drug Resistance Diagnosis System-CXR (TB-DRD-CXR). These methods were carried out in order to achieve the research objective.

### 3.1. Dataset and comparing method

This research utilized the Portal dataset, which can be obtained at http://tbportals.niaid.nih.gov (accessed on 2 September 2022). It was formerly employed in Rosenthal et al. (44) and Karki et al. (15). The dataset included 5,039 CXR images associated with tuberculosis, comprising 1,608, 470, 2098, 108, and 755 images for DS-TB, DR-TB, MDR-TB, pre-XDR-TB, and XDR-TB, respectively. All CXRs used in this study were frontal, AP, or PA views with varying resolutions (206 × 115 to 4,453 × 3,719 pixels). The intensity ranges identified in the photographs varied as well, with 1,177 images with low dynamic ranges, with intensities in the range of 0–255, and 579 images exhibiting high dynamic ranges, with intensities in the range of 0–65,536. The dataset was divided into two groups: the training dataset (80%; 4,030 images) and the testing dataset (20%; 1,009 images).

### 3.2. Developing an effective CNN model to classify drug resistance types

**Figure 1:**
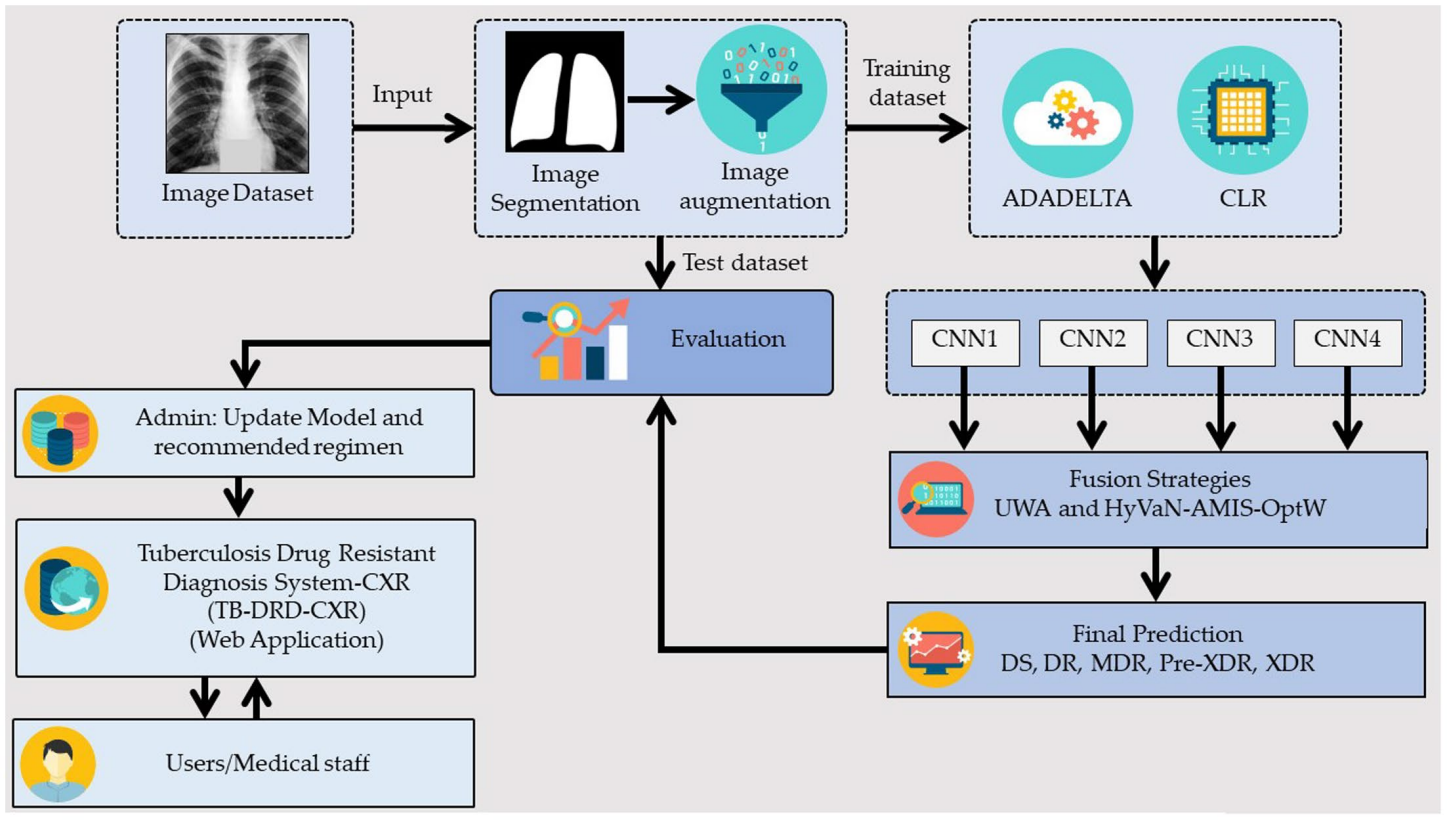
Generic framework of the proposed ensemble deep learning.

The generic model that is explained in this section is shown in Figure 1. The model was constructed using two preprocessing techniques, two decision fusion methodologies, and three distinct CNN architectures, as depicted in Figure 1. This research used two preprocessing techniques: (1) image segmentation, which was used to extract only the important part of the CXR, and (2) image augmentation, which was used to increase the quality and quantity of the input images. Later on, two learning rate strategies were applied. These two methods were an adaptive learning rate (ADADELTA) and a cyclical learning rate (CLR). Three effective CNN architectures were used to build the promising classification model. They were EfficientNetV2B3, EfficientNetV2M, and ConvNeXtSmall. Finally, two fusion strategies were applied to execute the final classification of DR.

#### 3.2.1. Image segmentation

The segmentation of the lungs employed in this study is a variant of that proposed in (42). By first breaking the original CXRs into smaller image patches, segmenting each one separately, and then piecing them back together, full segmentation can be achieved. This method employs a two-model ensemble, with the first model being a conventional convolutional neural network (CNN) that is used to categorize the image patches and then integrate them to generate a pre-segmentation. The second model is a custom version of the U-Net architecture that is used to perform the patch-based segmentation and then combines the results to produce a prior segmented image. The first segmentation is obtained by combining these two pre-segmented images via a binary disjunction operation; the final segmentation is obtained with postprocessing. Conventional image processing methods, such as erosion, dilation, connected component labeling, and region-filling algorithms, are used in the last steps of the process.

**Figure 2:**
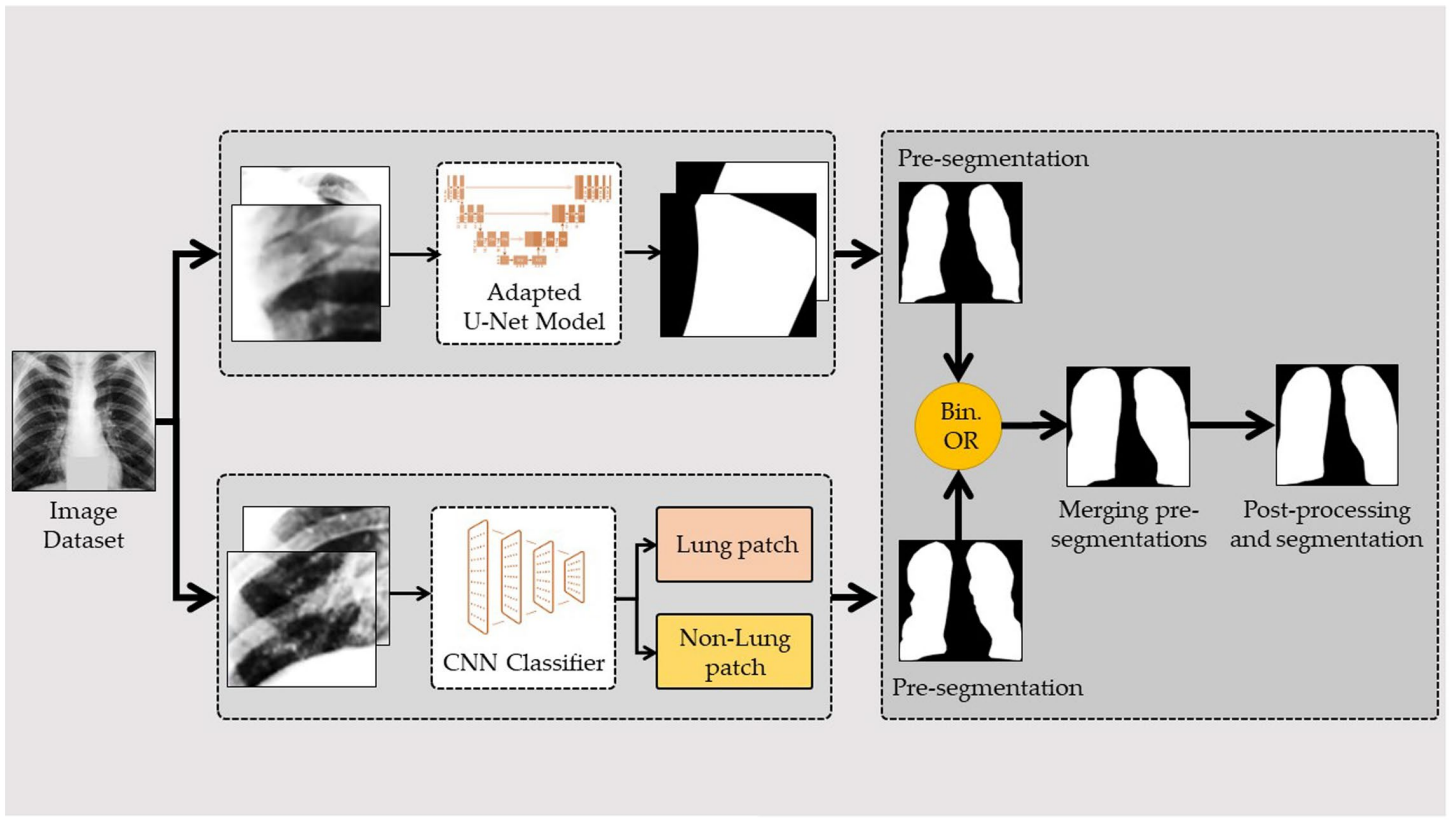
Framework of the lung segmentation.

Figure 2 illustrates a modified framework for lung segmentation developed from TB-Portal (44). Due to the fact that (in reality) CXRs may originate from various sources using various technologies, the quality of the image transmitted between the lab and the physician may vary. Therefore, good image preparation is needed to obtain input data with a steady quality. For image preparation, downscaling, resizing, normalizing, and extract patching were applied. For the U-Net architecture (31), the patch size needed to be large enough to be compatible with the downsampling and upsampling blocks. Therefore, we picked a 64*64 patch size for the U-Net layer. Similarly, while cropping the patches from the X-ray images, the patches from the respective masks were also cropped. This was performed to maintain the ground truth for each patch in the supervised training procedure. The CNN model was used to divide the image into lung and no-lung regions after the preprocessing steps had been completed. During the process of extracting the patches from the original X-ray images, they were categorized as either lung or nonlung. To accomplish this, patches were successively cropped from identical locations in the original X-ray and mask images. The lung-to-nonlung pixel ratio was then determined by comparing the cropped regions. If at least 20 % of the pixels in a given area were lung, we classified it as lung. It would not be dubbed a lung patch if it did not alleviate lung difficulties. Multiple experiments led to the empirical conclusion of a 20% lung patch threshold. After cropping and labeling the image, a customized CNN was utilized to identify CXR patches.

#### 3.2.2. Image augmentation

**Figure 3:**
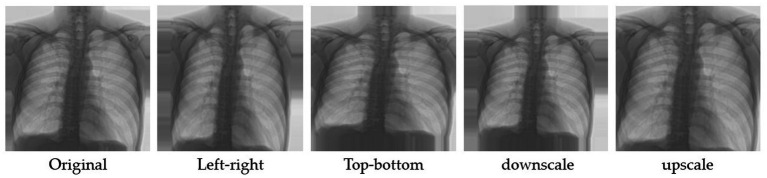
Example of left–right, top-bottom and linearly scaled method.

Image augmentation is a method for generating more training data for a model via the modification of existing data. In other words, it is the technique of artificially enlarging a dataset used to train a deep learning model. Using the following data augmentation approaches, the number of images within the employed datasets was increased in this study. The first group (A1-Aug) in image augmentation were random reflections of the original images in the left-to-right and top-to-bottom directions. The images were then linearly scaled along both axes using two random variables taken from a uniform distribution (45, 46). Figure 3 depicts an illustration of A1-Aug-type data augmentation.

Image rotation, translation, and shear made up the second set of data augmentation techniques, which are denoted as “A2-Aug.” The angle of rotation was decided at random and could be anywhere between −10 and 10 degrees. The translation shifted along both axes by a value that was randomly chosen from an interval that ranged from 0 to 5 pixels. Random samples were taken from the range [0, 30 degrees] for both the vertical and horizontal shear angles. The method of principal component analysis (PCA) that was described in Nanni et al. (47) was used for the third group in data augmentation, which is denoted as A3-Aug. Only the training data were used during the PCA space construction process. Three perturbation approaches were used to modify the PCA coefficients reflecting the original image vector; these perturbations yielded a new feature vector and, as a result, a new image once the perturbed vector was rebuilt. The first perturbation technique involved setting each element of the feature vector to zero at random (with a chance of 0.5). Five images were chosen at random from the same class as the original image for the third perturbation procedure. All six images were PCA-transformed, and some of the original image’s components were swapped with their matching components from the five remaining feature vectors. Each element of the five images had a 0.5% chance of replacing the original element. Since each color image had three channels, each channel was perturbed separately. This method augmented each original image with three additional images.

#### 3.2.3. Learning rate optimization

When training a convolution neural network (CNN), the learning rate hyperparameter is crucial. Adaptive learning rate algorithms seek to automate the process of figuring out the appropriate learning rate, as doing so manually remains a time-consuming operation. Two learning rates were employed in this study to enhance the precision of the classification. The first strategy was based on the work of ADADELTA (48). ADADELTA is an innovative gradient descent approach that accounts for learning rates in each dimension. Using only first-order data, the method dynamically adjusts over time, with very little additional processing burden compared to a standard stochastic gradient descent. The approach seems to be resilient in the face of noisy gradient information, alternative model architecture choices, diverse input modalities, and the selection of hyperparameters, and it does not necessitate any manual adjusting of the learning rate. ADADELTA outperformed competing single-machine and distributed cluster algorithms on the MNIST digit classification problem and a large-scale speech dataset. To avoid the drawbacks associated with using a fixed or exponentially falling value throughout training, Smith (49) proposed a cyclical learning rate (CLR) that changes within a range of values. The learning rate oscillates between its critical values as it follows the shape of a triangle window, Welch window (parabolic), or Hann window (sinusoidal). The rate of assimilation varies in a triangular window. The critical values are determined in advance. However, the CLR may momentarily lower the accuracy, despite being computationally easier than adaptive learning rates.

#### 3.2.4. CNN architectures

This study used three CNN architectures: EfficientNetV2B3, EfficientNetV2M, and ConvNeXtSmall. These three architectures were heterogeneously assembled into a single model, which was used to classify the type of medication resistance in patients based on their CXR images. We intended to develop an ensemble model with high precision and a low computational time. Using large CNN architectures increases the likelihood of achieving high accuracy (50). A combination of small, medium, and large architectures could produce an excellent categorization result, according to Xie et al. (51). However, in our suggested model, we selected the effective small CNN architecture as the base architecture and two effective large CNN architectures to increase the ensemble model’s performance.

DenNet-121 surpassed mobileNetV2 and ResNet101 in discovering the correct classification of drug resistance when combined with ensemble deep learning, according to Prasitpuriprecha et al. (21, 22). According to Chollet et al. (52), among all existing CNN architectures with a model size of less than 60 MB, EfficientNetV2B3 has the highest level of accuracy. It provides a 3.792% better answer than DenNet121, which corresponds to the outcome found in Liu et al. (53). Therefore, for the small CNN architecture, EfficientNetV2B3 was selected. Due to our desire to lower the computing time of the ensemble model, we restricted the size of the chosen model to no more than 250 MB for CNN architectures with larger sizes. According to Chollet et al. (52), the accuracy to model size ratio can be ranked from highest to lowest (top five highest ratios) as follows: ConvNeXtSmall (0.427), EfficientNetV2M (0.387), InceptionResNetV2 (0.373), ResNet152V2 (0.336), and ResNet152V2 (0.336). Incorporating the top two highest ratios into the suggested model was, therefore, the next step. The selected CNN architectures have the following details.

A new family of convolutional networks, the EfficientNetV2 series, outperforms its predecessor, EfficientNet version 1, in both training time and parameter efficiency. These models were created using a mixture of training-aware neural architecture search and scaling to simultaneously improve the training throughput and parameter efficiency. EfficientNetV2 uses an enlarged search space that includes novel operations such as Fused-MBConv to locate models. Experiments published in (54) demonstrated that EfficientNetV2 models train considerably faster than contemporary models while being up to 6.8 times smaller. Increasing the image size during training can help to accelerate the training process but often results in a loss of precision.

EfficientNetV2 suggests an enhanced approach to progressive learning that automatically modifies regularization as the image size increases to counteract this accuracy reduction. An EfficientNetV2 that was trained using reinforcement learning achieved dramatic improvements in performance over prior models on the ImageNet and CIFAR/Cars/Flowers datasets. An EfficientNetV2 that was pretrained on the same ImageNet21k dataset achieved 87.3% top-1 accuracy on the ImageNet ILSVRC2012 dataset, beating the latest ViT by 2.0% while training 5x-11x faster using the same computer resources. EfficientNetV2B3 has a size of 59 MB, while EfficientNetV2M has a size of 220B. The ConvNeXtSmall architecture, which is also utilized in this research, has a size of 192.29 MB.

**Figure 4:**
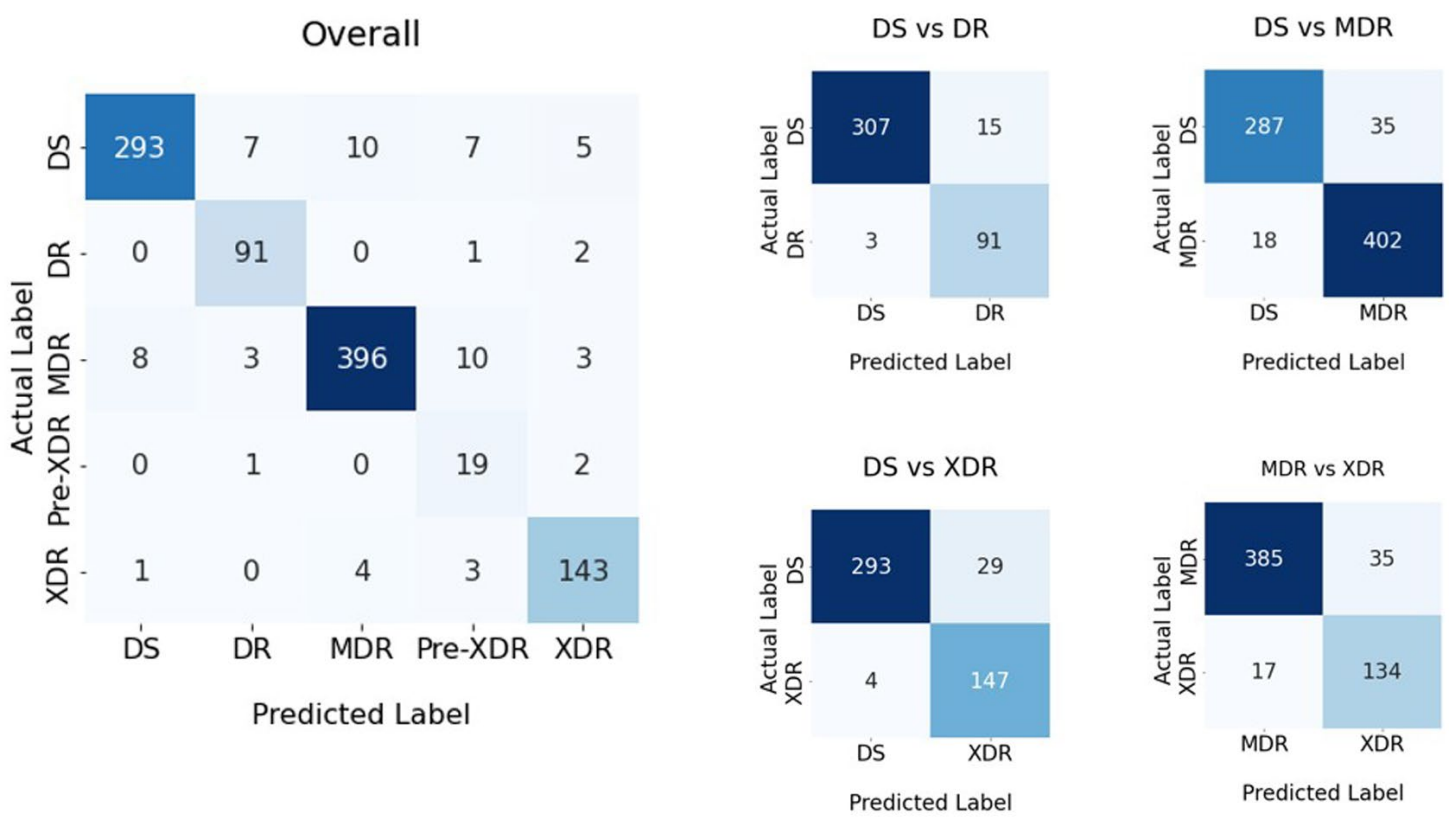
Confusion matrices of the classification result shown in Table 1.

ConvNeXtSmall (53) is a state-of-the-art image classification model. A vanilla vision transformer (ViT), on the other hand, faces difficulties when applied to general computer vision tasks such as object detection and semantic segmentation. It was the hierarchical transformers that reintroduced several prior ConvNets, making transformers practically viable as a generic vision backbone and demonstrating remarkable performances on a wide variety of vision tasks. ConvNet re-examines the design spaces and tests the limits of what a pure ConvNet can achieve. Constructed entirely from standard ConvNet modules, ConvNeXts compete favorably with transformers in terms of accuracy and scalability, achieving 87.8% ImageNet top-1 accuracy and outperforming transformers in COCO detection and ADE20K segmentation while maintaining the simplicity and efficiency of standard ConvNets. Figure 4 shows the architecture of a ConvNeXt.

#### 3.2.5. Decision fusion strategy

In this phase, the hybrid variable neighborhood strategy adaptive search (VaNSAS) (55) and artificial multiple intelligence system (AMIS) (56) (HyVaN-AMIS) was used to determine the proper fusion weight approach. A decision fusion strategy (DFS) handles combining the solutions from many CNN architectures into a single solution that represents the proposed model’s solution. First, the unweighted average model (UAM) was used to combine the answers. Then, HyVaN-AMIS was used to decide the best weight for the UAM in order to improve the quality of the final solution.

Numerous studies on deep learning and machine learning have made substantial use of the well-known unweighted average model (UAM) (54, 57, 58). The key idea is to give each prediction value ( $Y_{ij})$ the same weight, where *I* is the CNN label and *j* is the prediction class. Further in this study, the HyVaN-AMIS was integrated with the UAM to provide the ideal weight for the final forecast (HyVaN-AMIS-OptW).

The UAM used Eq. 1 for the fusion operations, while HyVaN-AMIS-OptW used Eq. 2 to determine the final weight. Let us define $Y_{ij}$ as the predicted value of CNN *i* class *j* prior to the application of Eqs 1, 2 $.V_{j}$ is the value used to classify class *j* following the fusion of multiple CNN results. $W_{i}$ is CNN i’s weight. I is the quantity of CNNs utilized by the ensemble model.


(1) $$V_{j}=\frac{\sum_{i=1}^{I}Y_{ij}}{I}$$

(2) $$V_{j}=\overset{I}{\underset{i=1}{\sum }}W_{i}Y_{ij}$$

The prediction is carried out by the class *j* with the highest value, as determined by the variable $V_{j}$ . The optimal value of $W_{i}$ is determined with the help of HyVaN-AMIS-OptW. HyVaN-AMIS-OptW is a process with five stages: (1) generating an initial set of tracks (Trs), (2) selecting an improvement box (IB), (3) having all tracks execute the chosen IB, (4) updating heuristics data, and (5) repeating stages (2)–(4) until the termination condition is met. In this study, the number of iterations serves as the model’s termination condition. *D* is the dimension of the *Tr*, which equals the number of CNNs (I). *NP* is the number of Trs that are constructed. An example of the Trs when M = 5 is {0.4,0.4,0.7,0.8,0.9}.

Each Tr makes a separate decision for which IB will be used to improve the current solution. As can be seen in Eq. 3, there is a clear probability function associated with choosing which Tr to employ. The IB is be chosen by the Tr in each repeat using a roulette wheel approach (46).


(3) $$P_{bt}=\frac{FN_{bt-1}+\left(1-F\right)A_{bt-1}+KU_{bt-1}}{\sum_{b=1}^{B}FN_{bt-1}+\left(1-F\right)A_{bt-1}+KU_{bt-1}}$$

The probability of IB *b* in iteration *t* is denoted by $P_{bt}$ . *F* is the scaling factor, which is set to 0.7 according to the recommendation in Ganaie et al. (23). $A_{bt-1}$ is the average objective function of all Trs that chose IB *b* from iteration 1 to iteration *t*-1. *K* is a predetermined integer value set to 3 (23). $U_{bt-1}$ is a positive integer that rises by 1 if the iteration’s best solution is in IB *b*. $N_{bt-1}$ is the total number of Trs that selected IB *b* from iteration 1 to iteration *t*-1, each of which has to be iteratively updated.

$P_{bt}$ is the probability of IB *b* occurring at iteration *t*. Scaling factor F was recommended by Pitakaso et al. (56) to be 0.7. The objective function $A_{bt-1}$ is the weighted average of all Trs’ objective functions from iteration 1 through *t*-1 when they choose IB b. *K* is an unchanging integer, and it was set to 3 (56). If the best solution of the iteration is in IB *b*, then $U_{bt-1}$ increases by 1. The total number of iterations from the first iteration to iteration *t*-1 in which Tr chose IB b is denoted by $N_{bt-1}.$ Each of these variables must be iteratively updated. Once the Tr has determined which IB will be used to improved itself, it will make that decision using the probability function provided in Eq. 4. Three improvement boxes, as listed in Table 2, were used in this research (Eqs 4–6), as recommended in Pitakaso et al. (55, 56).


(4) $$Z_{elt}=\rho X_{rlt}+F1\left(B_{l}^{gbest}-X_{rlt}\right)+F2\left(X_{mlt}-X_{rlt}\right)$$

(5) $$Z_{elt}=X_{rlt}+F1\left(B_{l}^{gbest}-X_{rlt}\right)+F2\left(B_{el}^{pbest}-X_{rlt}\right)$$

(6) $$Z_{elt}=X_{rlt}+F1\left(X_{mlt}-X_{nlt}\right)$$

The IB equations are identical to the equations shown in Pitakaso et al. (55, 56), and they were designed in accordance with multiple intelligence theory. $X_{elt}$ is the value of Tr *e* element l in iteration *t*, whereas *r*, *n*, and *m* are non-equal elements of Tr (1 to E) not equal to *e*. $B_{l}^{gbest}$ is the global best Tr, i.e., the Tr that delivers the best solution out of all generated solutions (iteration 1 to the current iteration).

$H_{el}$ represents a random number of Tr *e* element *l*. F1 and F2 are the scaling factors, which are defined as 3 [as recommended by (45)], and CR is the crossover rate, which Pitakaso et al. (56) suggested is equal to 0.8. $R_{elt}$ is a Tr produced arbitrarily from Tr *e* element *l* during iteration *t*. Tr *e* discovers $B_{el}^{pbest}$ to be the best answer. Eq. 7 is used to update the value of $X_{elt+1},$ and $X_{elt+1}$ is related to the values of $W_{i}$ , as indicated in Eq. 8.


(7) $$X_{elt+1}=\{\begin{array}{c} Z_{elt}\;if\;f\left(Z_{elt}\right)\leq f\left(X_{elt}\right)\;andupdate\;f\left(X_{elt}\right)=f\left(Z_{elt}\right)\\ X_{elt+1}\;otherwise\; \end{array}$$

(8) $$W_{i}=X_{eit}\;\mathrm{for}\;\mathrm{all}\;e\;\mathrm{and}\;t.$$

This paper investigated image segmentation (Seg), augmentation (Aug), ensemble deep learning with a heterogeneous ensemble architecture, two learning rate techniques, and decision fusion procedures. Table 1 depicts the experimental design of the recommended methods for classifying the medicine resistance level. Nevertheless, as seen in Table 3, this study included 16 experiments.

**Table 1:** Accuracy of the classification using different strategies in fast image analysis.

	Number of input images	Number of correct classification	% Correct classification	Number of wrong classification	% Wrong classification
Strategy 1	414	378	91.3	36	8.7
Strategy 2	414	403	97.3	11	2.7
Strategy 3	414	401	96.9	13	3.1
Strategy 4	414	380	91.8	34	8.2

### 3.3. TB drug resistance diagnosis system-CXR (TB-DRD-CXR) design

**Figure 5:**
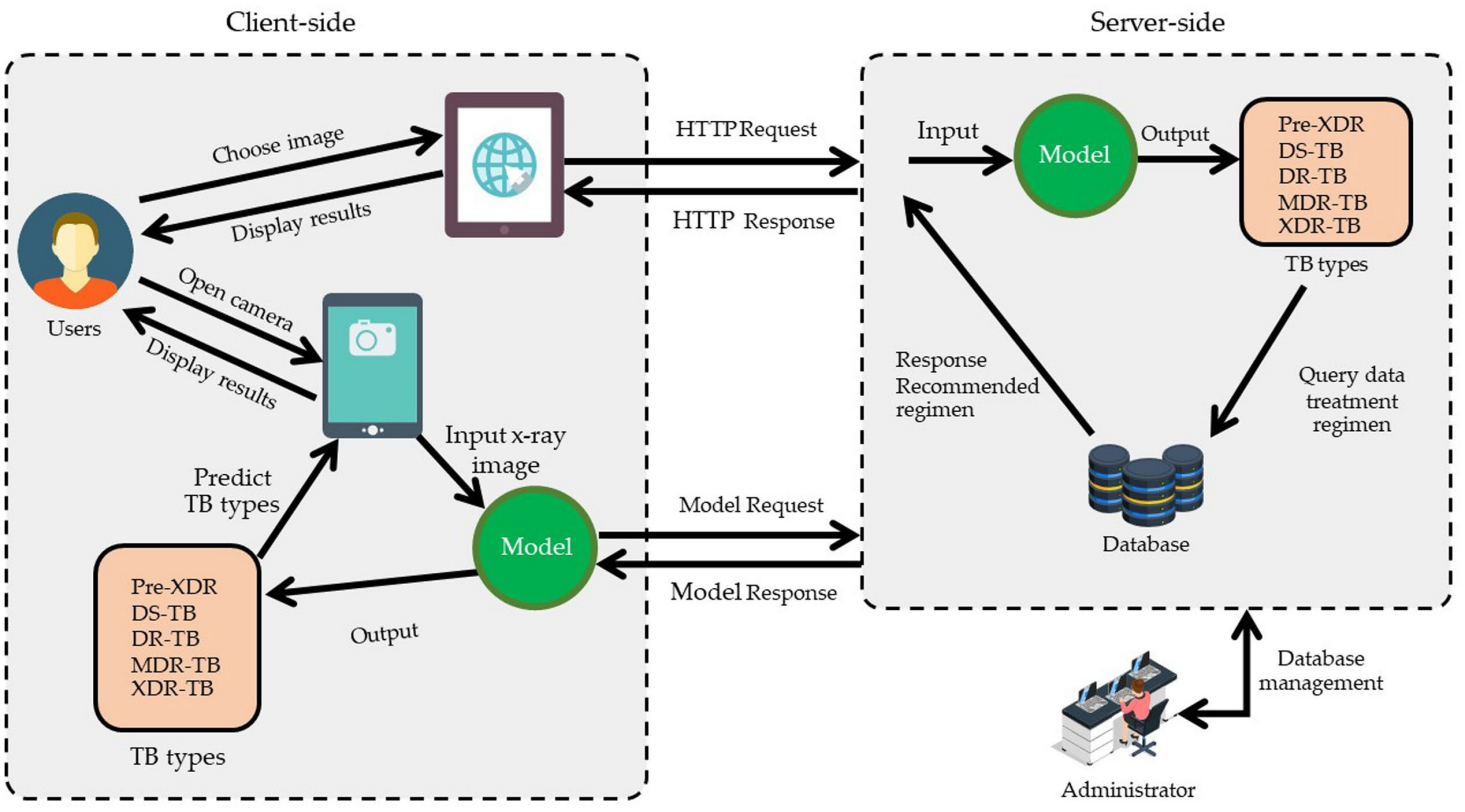
The design framework of web application for the case study.

TB-DRD-CXR was designed to help medical staff in the drug resistance diagnosis process. Web applications are programs that may be accessed and used exclusively online. Online programs are hosted on a remote server, as opposed to being downloaded, installed, and run locally on the user’s machine. In order to view them, all you need is a computer equipped with a web browser and a connection to the internet. To improve the usability of a website or web application for its visitors, professional web designers focus on the interface as well as the experience they have there. Web design is distinct from software development because it focuses on the website’s functionality, accessibility, and esthetics. This system’s web application architecture design is shown in Figure 5 below.

**Figure 6:**
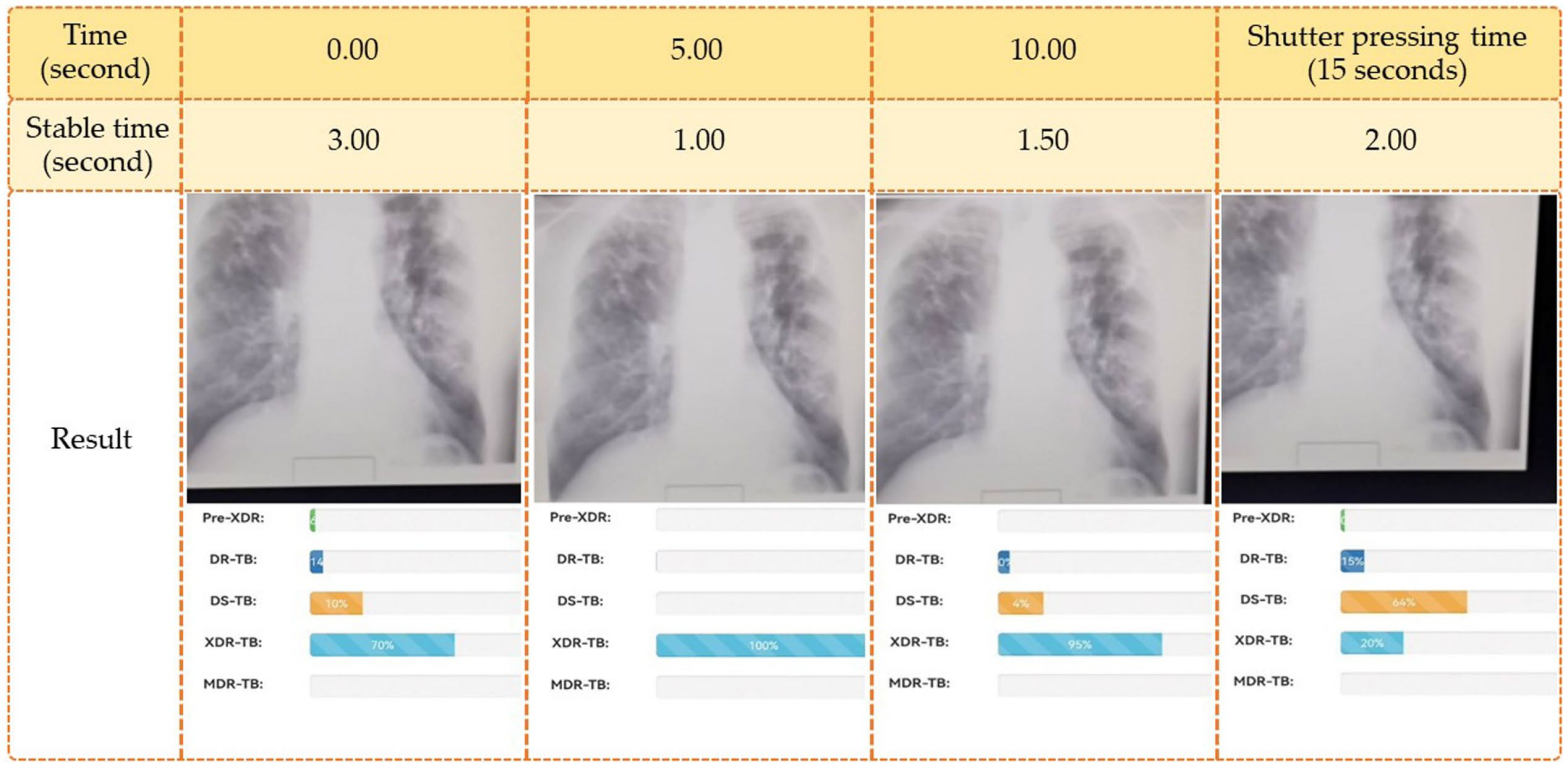
Example of the live camera (fast image analysis).

According to Figure 5, the web application was built using HTML, JavaScript, and TensorFlow, adhering to the principles of responsive design so that it would work properly on any modern web-enabled device. It is possible for users (medical professionals) to submit an image file for further analysis on their own PCs. A server receives this image file in order to make a diagnosis and provide medication recommendations. The pharmacist who handles this matter updates the treatment plan. Once a picture is obtained, the system uses a deep learning model to make a diagnosis as to what ailment is present. Once the disease classification is determined, it returns to the user’s side with specific information and recommendations regarding treatment options. The users’ interface that we used in this research was composed of two ways to insert images into the system for diagnoses. These two methods were selecting an image from the sources in the computer and via a live insertion of an image (fast image analysis). For the selecting image input, the result was displayed once to the user when the operating system had completed classifying the drug resistance type. The live input data reported the outcome multiple times throughout the live image input process. The live input procedure concluded when the user pressed the camera’s shutter button. The reporting strategy for the live camera segment consisted of four tactics, including (1) reporting the last classification result when the user pressed the camera’s shutter; (2) reporting the class of drug-resistant organisms with the highest likelihood during the entire live camera session; (3) reporting the class of drug-resistant organisms with the longest stable likelihood during the live camera session; and (4) reporting the class with the highest average likelihood during the live camera session. Table 4 illustrates an example of a class decision for a live camera, whereas Figure 6 illustrates an example of the user interface for a live camera.

Using strategies (1), (2), (3), and (4), as shown in Figure 6, the final decision classes were DR-TB, MDR-TB, XDR-TB, and MDR-TB, with probabilities of 79, 100, 82, and 46%, respectively.

**Figure 7:**
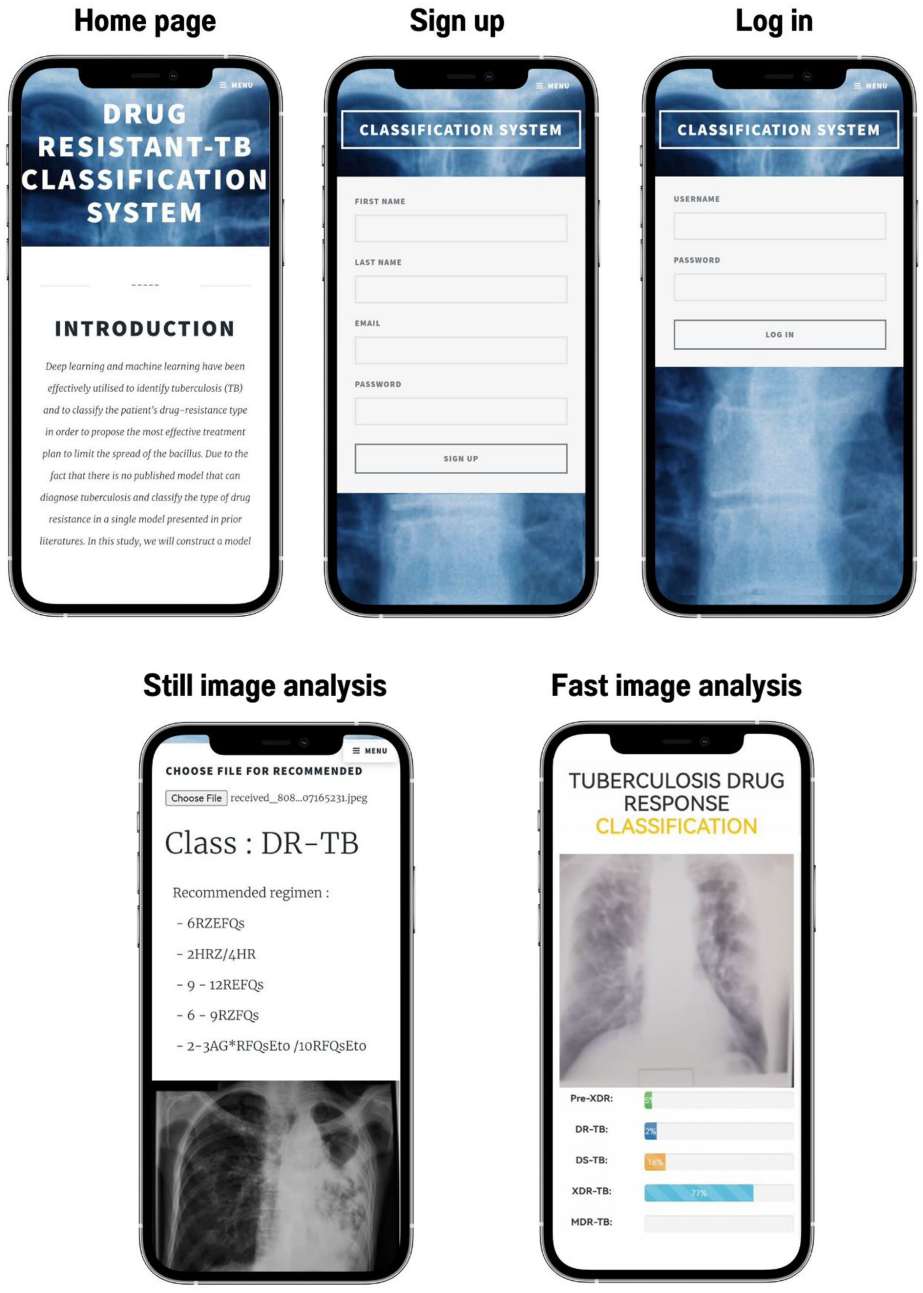
User interface of TB-DRD-CXR.

Figure 6 demonstrates that if various methods are employed to forecast the final drug resistance class using the live camera, the outcome is entirely different. In this section, however, we randomly selected 414 images from the Portal dataset in order to determine which of the proposed classification algorithms had the highest accuracy. This strategy was then utilized to categorize drug-resistant patients within the proposed application. The method analyzed the system’s efficiency, the quality of the user experience, and the likelihood that consumers would employ TB-DRD-CXR as a tool to aid in the medical staff’s TB drug resistance diagnosis decision making. An example of the user interface is shown in Figure 7.

The choice of usability measures for the study was based on ISO 9241-11:2018 (63), an updated version of ISO9241 (64). The methodology that we used to reveal the effectiveness of the application was the method that was used in Escanillan-Galera et al. (65). It was suggested that the usability measures should be effective, efficient, and comfortable. These measures were chosen because the properties of mistakes from the model in (64) were related to efficacy. The participants were medical personnel from the 14 large hospitals in northeastern Thailand. In total, 33 physicians attempted to employ TB-DRD-CXR.

The participants were users of both mobile devices and computers who ranged in age from 28 to 58. Before beginning the usability test, the participants were given forms and instructions outlining what would take place. The researcher was also available to address any questions participants might have had. An assortment of assignments was handed out to the participants. Since TB-DRD-CXR only allows one user account per medical staff, they had to complete the required steps one at a time. The participants utilized a designated smartphone or a computer that was online.

**Figure 8:**
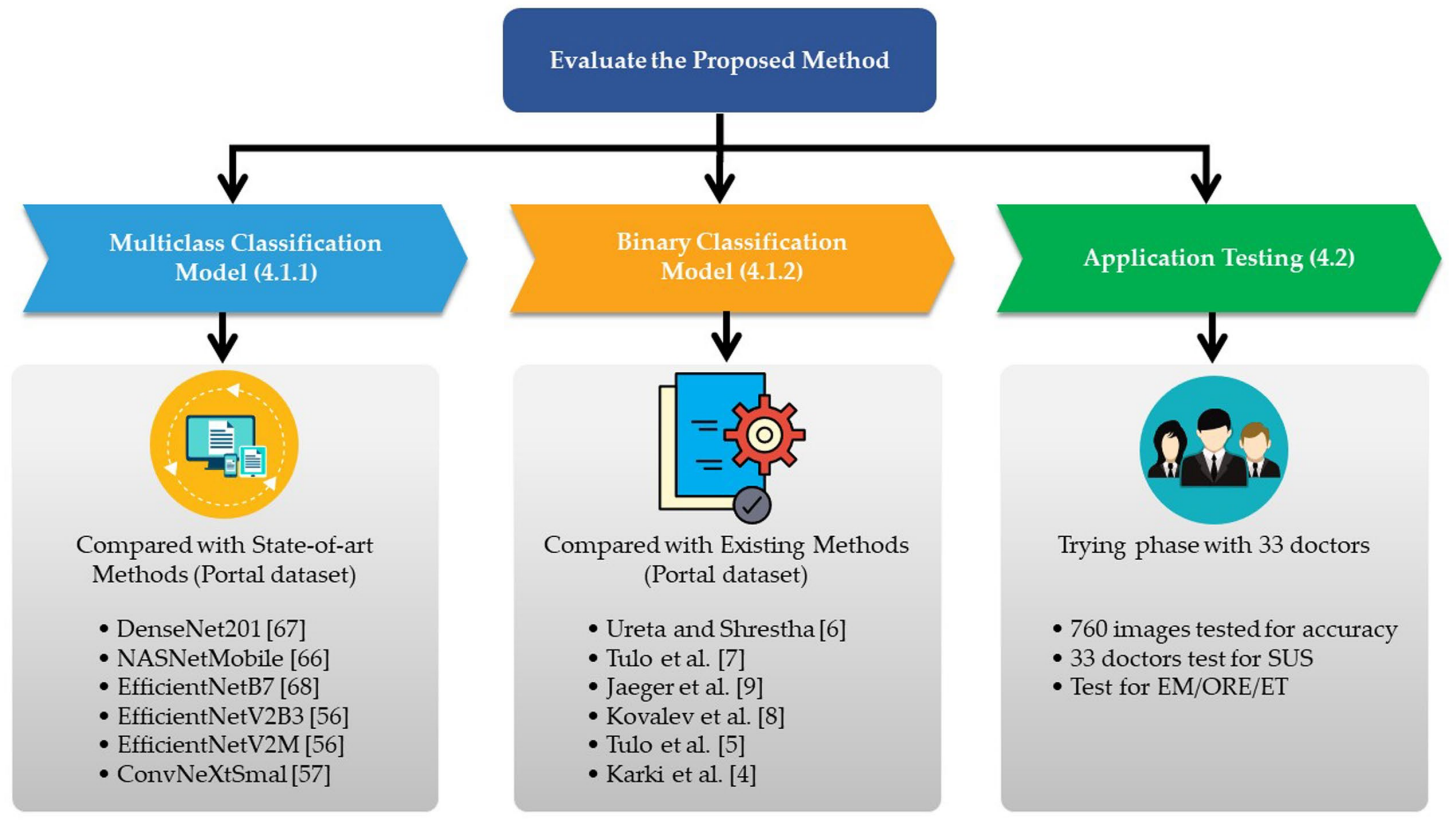
Experimental framework of the proposed model & application.

Users had already been provided CXR images prior to the test via the app. Using the system usability scale (SUS) form created by Brooke (65), the participants rated their level of contentment and overall experience with the mobile web application. Each trial participant was given a separate evaluation sheet that included ten (10) different tasks that required them to execute a task in TB-DRD-CXR. The test results for the user interface are shown in Figure 8. Ten minutes were allotted for each person to fill out the evaluation form. The success rate was calculated by tallying the number of objectives that were met. Examples of the tasks include “Open browser and go to https://itbru.com/TDRC/index.html” and “Select to detect by selecting the image from computers/mobile.” The effectiveness of the model (EM) was calculated using Eq. 9.


(9) $$\begin{array}{l} EM=\\ \frac{Totalnumberoftasksthatcompleted\;by\;all\;participants\;}{Totalnumberoftasks}\times 100\% \end{array}$$

The effectiveness of the program was evaluated based on how quickly the task could be completed by the user. Eq. 10 demonstrates how to do so in terms of time-based efficiency (ET), and Eq. 11 demonstrates how to do so in terms of overall relative efficiency (ORE).


(10) $$ET=\frac{\sum_{j=1}^{J}\sum_{i=1}^{I}\frac{n_{ij}}{t_{ij}}}{I\times J}$$

(11) $$ORE=\frac{\sum_{j=1}^{J}\sum_{i=1}^{I}n_{ij}t_{ij}}{\sum_{j=1}^{J}\sum_{i=1}^{I}t_{ij}}\times 100\%$$

$n_{ij}$ : user’s performance on task *i,* where J = total number of users and I = total number of tasks (j). $n_{ij}$ : 1 if the task is completed; otherwise, $n_{ij}$ : = 0. The amount of time it took for user j to finish the task is $t_{ij}$ . If the user quit before the work was finished, the clock stopped ticking at the time of their last action.

In the second experiment, the SUS score was determined by having participants rate 10 items on a 5-point scale ranging from 1 (“Strongly disagree”) to 5 (“Strongly agree”) (66). In total, there were 5 affirmative sentences and 5 negative ones. Each item’s score was added together, and the SUS score was determined. The weight of each factor in the total score varied from 0 (no weight) to 4 (high weight). To calculate a score, 1 was subtracted from the scale position for items 1, 3, 5, 7, and 9. The contributions of items 2, 4, 6, 8, and 10, with negative wordings, were 5 minus the scale position. An SUS value could be calculated by multiplying the sum of the scores by 2.5. The percentile scores on the scale used to rate the usability of the system varied from zero to one hundred. An acceptable SUS score scale was developed in Bangor et al. (67). In general, if the SUS sum is in the 85–100 zone, it means that customers enjoy using the system and are likely to recommend it to others. When the SUS score is in the range of 70 and 85, the usability of the system is very good. A score between 50 and 70 indicates that the system is generally acceptable but not without problems for its users. Finally, a score below 50 indicates that users did not enjoy using the system, indicating that there is a problem with the system that needs to be addressed. For the sake of this analysis, we employed this scale of suitable SUS scores. Examples of positive questions include “the user anticipates they will use this system regularly” and “the interface was straightforward,” while examples of negative questions include “the application seemed overly complicated and time-consuming” and “this app seems too inconsistent for my liking.”

## 4. Computational results and framework

In this part, we provide the results of our computational analysis in two parts: the results of (1) an experiment to prove the viability of the proposed methods and (2) the effectiveness of the development of a web application for the categorization of preventable adverse drug reactions. All experiments were executed using a PC that had an Intel i7 2.1 GHz (8 core) CPU, 32 GB of RAM, and a Tesla V100 GPU (16 GB of GPU RAM). The experimental framework is shown in Figure 8.

### 4.1. Evaluating the performances of the suggested approaches

In Section 4.1.1, we determine which of the possible combinations shown in Table 2 is the best way to classify the type of medication resistance of a patient. In Section 4.1.2, we evaluate the suggested model’s efficacy in light of the existing heuristics.

**Table 2:** KPIs result in comparing with the state-of-art methods.

CNNs architecture	KPIs			Model size (MB)	Model Size (6 CNN)	Training time (minutes)	Testing time (seconds per dataset)
	AUC	F-Measure	Accuracy				
DenseNet201 (59)	75.4	73.8	72.5	80	480.00	240.7	30.3
ResNet101 (60)	72.8	71.2	69.4	171	1,026	512.5	68.7
NASNetMobile (61)	52.7	50.4	48.3	23	138.00	80.5	10.8
EfficientNetB7 (62)	93.1	91.9	90.7	256	1,536.00	778	42.1
EfficientNetV2B3 (54)	67.4	65.4	63.1	59	354.00	210.7	94.5
EfficientNetV2M (54)	92.8	90.4	89.5	220	1,320.00	684.5	78.4
ConvNeXtSmal (57)	90.3	87.9	86.8	192.29	1,153.74	601.2	68.9
Proposed Method	95.1	94.8	93.4	130.26	781.58	301.4	38.4

**Table 3:** Detail of the 16 experiments.

#Experiment	No Seg	Seg	No Aug	Aug	ADADELTA	CLR	UWA	HyB
1	√		√		√		√	
2	√		√		√			√
3	√		√			√	√	
4	√		√			√		√
5	√			√	√		√	
6	√			√	√			√
7	√			√		√	√	
8	√			√		√		√
9		√	√		√		√	
10		√	√		√			√
11		√	√			√	√	
12		√	√			√		√
13		√		√	√		√	
14		√		√	√			√
15		√		√		√	√	
16		√		√		√		√

**Table 4:** AUC, F-Measure, and accuracy of 16 experiments utilizing Multiclass DR Classification.

#Experiment	AUC (%)	F-measure (%)	Accuracy (%)
1	67.4	63.8	65.1
2	68.8	65.9	66.4
3	70.3	68.3	67.8
4	71.4	70.2	68.3
5	73.5	67.4	69.4
6	75.9	72.9	71.7
7	77.3	71.3	73.0
8	79.6	77.8	75.8
9	83.5	80.1	81.1
10	87.4	85.4	83.9
11	88.9	86.9	85.4
12	89.4	87.8	87.3
13	90.5	89.3	88.9
14	92.9	91.4	90.2
15	93.5	91.9	91.7
16	95.1	94.8	93.4

Conclusion								
	No Seg	Seg	No Aug	Aug	ADADELTA	CLR	UWA	HyB
AUC	73.0	90.2	78.4	84.8	80.0	83.2	80.6	82.6
% Diff-AUC	23.6		8.2		4.0		2.5	
F-Measure	69.7	88.5	76.1	82.1	77.0	81.1	77.4	80.8
%Diff F-measure	27.0		7.9		5.3		4.4	
Accuracy	69.7	87.7	75.7	81.8	77.1	80.3	77.8	79.6
%Diff -Accuracy	25.8		8.1		4.2		2.3	

**Table 5:** The classification result of the proposed method comparing with various previous methods.

DS-TB vs. DR-TB							
Research	Type of classification	Classes	Features	Region in CXR	AUC	F-measure	Accuracy
Ureta and Shrestha (6)	Binary class	DS vs. DR	CNN	Whole	67.0	–	–
Tulo et al. (7)	Binary class	DS vs. DR	Shape	Mediastinum+ Lung		93.6%	
Kovalev et al. (8)	Binary class	DS vs. DR	Texture and Shape	Lung	–	–	61.7
Karki et al. (4)	Binary class	DS vs. DR	CNN	Lung excluded	79.0	–	72.0
The Proposed Method	Binary class	DS vs. DR	Ensemble CNN	Lung excluded	97.8	97.4	96.4
DS-TB vs. MDR-TB							
Jaeger et al. (9)	Binary class	DS vs. MDR	Texture, Shape and Edge	Lung	66%	61%	62%
Tulo et al. (5)	Binary class	DS vs. MDR	Shape	Mediastinum+ Lungs	87.3	82.4	82.5
The Proposed Method	Binary class	DS vs. MDR	Ensemble CNN	Lung excluded	94.1	93.5	92.8
DS-TB vs. XDR-TB							
Tulo et al. (5)	Binary class	DS vs. XDR	Shape	Mediastinum+ Lungs	93.5	87.0	87.0
The Proposed Method	Binary class	DS vs. XDR	Ensemble CNN	Lung excluded	94.9	94.7	93.1
MDR-TB vs. XDR-TB							
Tulo et al. (5)	Binary class	MDR vs. XDR	Shape	Mediastinum+ Lungs	86.6	81.0	81.0
The Proposed Method	Binary class	MDR vs. XDR	Ensemble CNN	Lung excluded	92.2	91.5	90.9

**Table 6:** TB-DRD-CXR accuracy result.

	Number of input images	Number of correct classification	% correct classification	Number of wrong classification	% Wrong classification
DS-TB	173	167	96.5	6	3.5
DR-TB	128	124	96.9	4	3.1
MDR-TB	196	187	95.4	9	4.6
Pre-XDR-TB	84	81	96.4	3	3.6
XDR-TB	179	171	95.5	8	4.5
Total	760	730	480.7	30	19.3
Average	152	146	96.1	6	3.86

#### 4.1.1. Evidence for the most promising alternative strategies

The experimental batch sizes for EficientNetV2B3, EficientNetV2M, and ConvNeXtSmall were 16, 2, and 8, respectively. To train the model, 200 epochs were used (68). The computational outcomes of all 16 experiments are shown in Table 4.

The % difference was calculated using Eq. (12). $Obj^{C}$ is the objective function (KPI) of the challenger method. $Obj^{D}$ is the objective function of the defender method. The defender methods are models that have no segmentation, have no augmentation, use ADADELTA, and use UWA. The % difference and average AUC, F-measure, and accuracy values of all methods are shown in Table 4.


(12) $$\%diff=\frac{Obj^{C}-Obj^{D}}{Obj^{D}}\times 100\%$$

Using the AUC, F-measure, and accuracy as the KPIs, Tables 1, 4, demonstrate that the image segmentation technique improved the quality of the solution by 23.6, 27.0, and 25.8% when compared to the solution that did not employ image segmentation. The image augmentation enhanced the accuracy by 8.1% compared to the control group. CLS provided 4.2% greater accuracy than ADADELTA, whereas HyVaN-AMIS provided 2.3% greater accuracy than UWA. The experiment that provided the highest AUC, F-measure, and accuracy values was experiment number 16, which used image segmentation, augmentation, CLR, and HyVaN-AMIS in the model. We used this model to compare with other methods found in the literature. A confusion matrix of the multiclass classification model is shown in Figure 4.

DS-TB, DR-TB, MDR-TB, and XDR-TB had accuracy values of 92.2, 97.1, 88.7, and 91.1 percent, respectively, as shown in Figure 4. There was a 0% mistake rate when classifying non-TB and DS-TB but 2, 1, and 1% error rates when the results were DR-TB, MDR-TB, or XDR-TB instead of non-TB. However, the prediction error for classes DR-TB, MDR-TB, and XDR-TB was greater than for classes DS-TB, XDR-TB, and DR-TB, respectively. The DS-TB prediction class had the greatest classification error if it was classified as MDR-TB.

The proposed methods were compared with the traditional homogeneous ensemble deep learning in the next experiment. The CNN architectures that were compared with the proposed methods included EfficientNetV2B3, EfficientNetV2M, ConvNeXtSmall, DenseNet201 (69), NASNetMobile (61), and EfficientNetB7 (59). All methods were executed for two hundred epochs using batch sizes of 2, 3, 5, 12, 10, and 8 for EfficientNetV2B3, EfficientNetV2M, ConvNeXtSmall, DenseNet201, NASNetMobile, and EfficientNetB7, respectively. The results of the comparison are shown in Table 2.

Table 2 demonstrates that the proposed strategy, which employs heterogeneous ensemble deep learning, as opposed to homogeneous ensemble learning, was superior. It was proven that the proposed method required a 125.2% longer training and testing time than DenseNet201, but the proposed methods provided 128.8% higher accuracy than DenseNet201; in other words, the proposed method increased the accuracy by 3.6% more than the increase in computational time. Compared to previous methods such as NASNetMobile and EfficientNetV2M, the suggested method increased the accuracy by 13.43% and the computing time by 3.47%. The proposed method was, on average, 30.6% more accurate than the state-of-the-art methods.

#### 4.1.2. Comparing the results with the existing drug resistance classification methods

The following experiment evaluated the efficacy of the proposed model compared to the state-of-the-art methods found in the literature by using the proposed model to classify a binary classification model and comparing it to the existing methods; the results are shown in Table 5.

We determined, based on the computational results, that when classifying DS-TB versus DR-TB, the proposed model was 33.9% more accurate than the existing method. The suggested approach improved the solution quality by 12.48% for identifying DS-TB versus MDR-TB compared to (5). The suggested approach increased the classification accuracy by 4.0% when comparing DS-TB to XDR-TB and by 12.2% when classifying MDR-TB and XDR-TB. However, we can infer that the proposed strategy beat all state-of-the-art methods reported in the literature for classifying the categories of drug-resistant patients.

### 4.2. The testing of TB-DRD-CXR

TB-DRD-CXR, which was designed utilizing the framework described in Section 3.2, will be tested by distributing the program to expert TB physicians. Once the TB-DRD-CXR system’s design is finished, three studies will be run with volunteers (users) to test its efficacy. To begin, 760 CXR images were chosen at random from the Portal dataset. Similar CXR images were used as inputs in a test to see how well TB-DRD-CXR can categorize CXR images. The test outcomes are summarized in Table 6.

**Figure 9:**
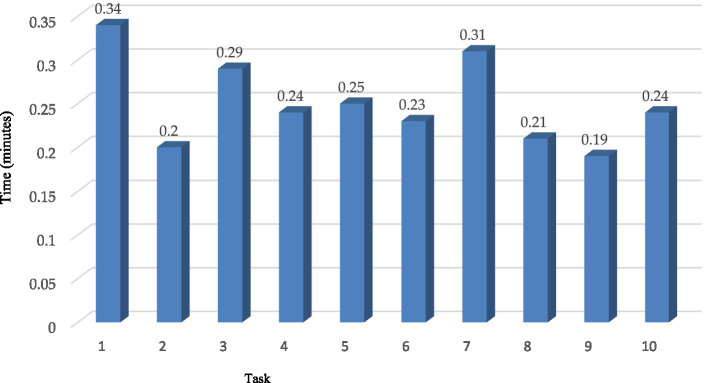
Average time used by all participants to execute each task.

Table 6 shows that the suggested method had a 96.1% success rate in testing in a real implementation, resulting in just a 3.86% failure rate when classifying TB-DRD-CXR images. The next phase of TB-DRD-CXR testing focused on the reaction of users to the device. In the next stage of the study, TB-DRD-CXR testing was conducted. An assignment was given to 33 participants. The results of the assignment showed that the average time it took for all 33 participants to finish all 10 activities was 7.57 min. The time-based efficiency (ET) yielded a goal/min value of 4.16 and an overall relative efficiency (ORE) of 100% when assessing the mobile web application, with a goal/min value of 4.16 indicating high relative efficiency. The typical time required to complete each activity is depicted in minutes in Figure 9.

According to Figure 9, “opening the application” was the most time-consuming task for all users, requiring 0.34 min. “Finding the CXR from their computers” took 0.29 min but could take longer if the user could not recall where they saved the images. The shortest amount of time needed to complete a task was 0.20 min; this was the amount of time it took to select a browsed image, which required only one click to select. In total, 12 out of 33 people had an SUS score of 100 percent using TB-DRD-CXR, according to the computed result, which indicates that 52 percent of participants appreciated the suggested application completely. In addition, 100% of the TB-DRD-CXR scores were greater than 90 SUS points. TB-DRD-CXR had an overall SUS score of 96.7, which indicates that customers enjoyed using the system and are likely to recommend it to others, per the suggestion in (67).

In the final research study, we used four different methods to determine how the quick image analysis system’s classification results should be interpreted. We provided four strategies: (1) reporting the last classification result when the user presses the camera’s shutter; (2) reporting the class that has the highest likelihood during the whole live camera session (LCS); (3) reporting the class with the longest stable likelihood during the LCS; and (4) reporting the class with the highest average likelihood during the LCS. A set of 414 images from the Portal dataset were chosen at random and checked for accuracy. Table 1 shows the results of the experiments.

Based on the information in Table 1, we can conclude that the optimal method for determining the result of the fast image analysis mode is strategy 2, which reports the drug resistance type with the highest probability throughout the whole live camera session. Because of this method, the AI should be able to extract the best result possible from the current image.

Explainable AI (XAI) techniques, such as Grad-CAM (62), have become increasingly important in improving the interpretability and trustworthiness of deep learning models. Grad-CAM is a technique utilized to generate heatmaps that explain the predictions made by deep learning models, such as the one employed in the TB-DRD-CXR application for classifying different types of drug-resistant tuberculosis (TB) using chest X-ray images. By capturing the gradient information of the model’s output with respect to the final convolutional layer of the network, Grad-CAM identifies and highlights the regions in the input image that had the most significant influence on the prediction, visualized as heatmaps with brighter areas indicating higher importance.

In the context of the TB-DRD-CXR application, the use of Grad-CAM heatmaps provides valuable insights into the important regions in the chest X-ray image that influenced the model’s classification of various drug-resistant TB types. These heatmaps provide visual explanations of the model’s decision-making process, enhancing the interpretability and understandability of the predictions, resulting in improved transparency, explainability, and trustworthiness of the application. By incorporating Grad-CAM heatmaps into the TB-DRD-CXR application, users can gain a better understanding of the model’s decision-making process, enabling better decision-making in the context of TB classification and enhancing the overall usability and effectiveness of the application for identifying different types of drug-resistant TB cases.

**Figure 10:**
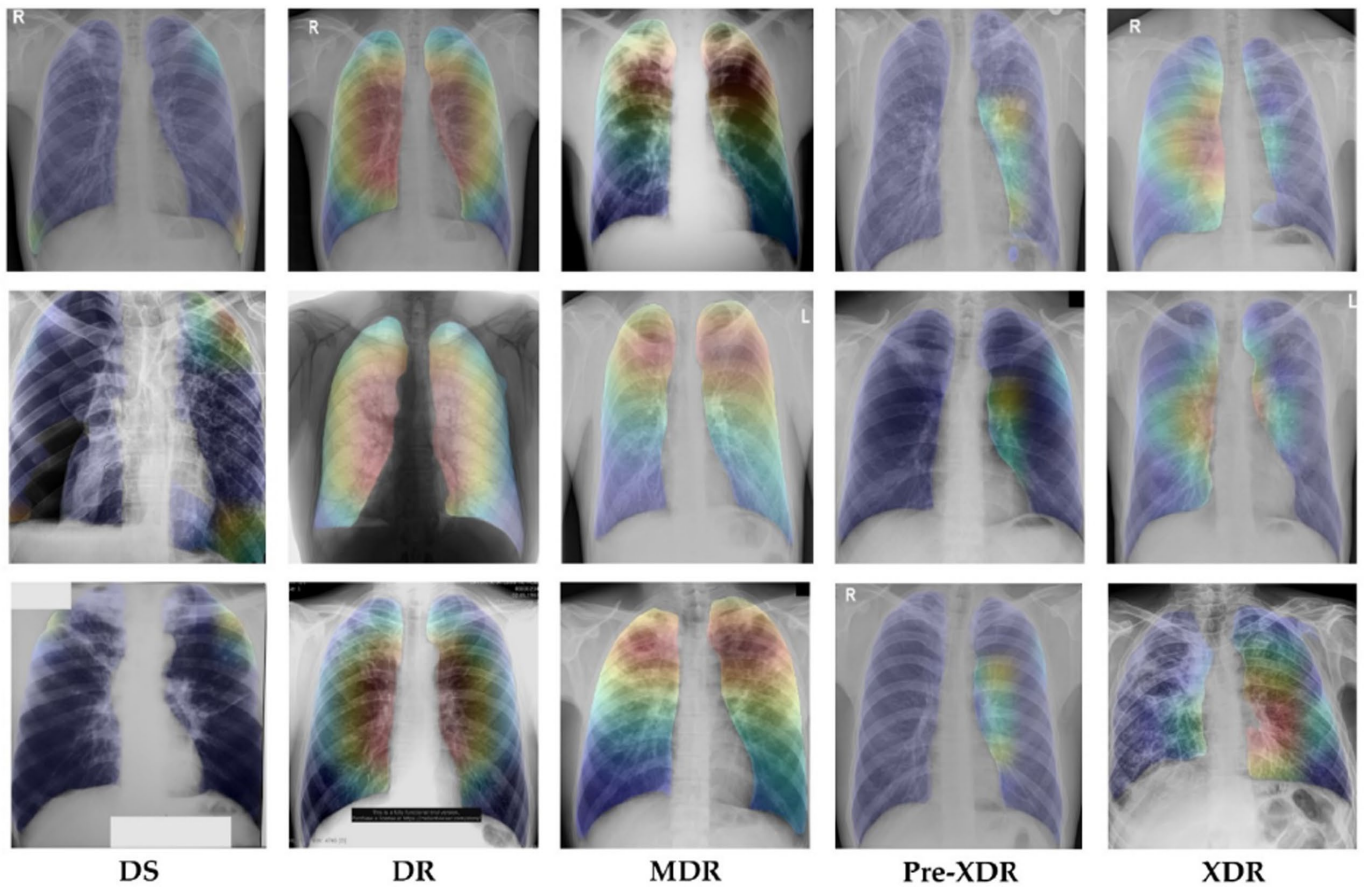
Heatmap of the classification model.

Figure 10 showcases the application of Grad-CAM heatmaps to visualize the classification of different drug-resistant types of tuberculosis, providing a clear and interpretable representation of the model’s classification process. The developed model utilizes all six areas of the lung for non-TB classification, while focusing on specific lung sectors for identification of different drug-resistant TB types. The use of Grad-CAM heatmaps in this study confirms the accuracy of the model’s decision-making process, verifying the reliability of its predictions. As illustrated in Figure 10, the model demonstrates a high degree of accuracy in identifying the relevant regions, effectively highlighting the innermost area of the lung for DR-TB, the upper regions of both left and right lungs for MDR-TB, the middle to lower innermost area of the lung for Pre-XDR-TB, and the lower innermost areas of both left and right lungs for XDR-TB. These findings unequivocally affirm the effectiveness of the model in accurately identifying various types of drug-resistant TB cases. The heatmap and accompanying explanation are coherent, enhancing trust in the interpretability of the proposed model and addressing concerns regarding the opacity of deep learning models. The use of Grad-CAM heatmaps in this study demonstrates the value of Explainable AI (XAI) techniques in improving the interpretability and trustworthiness of deep learning models, enabling better decision-making in the context of TB classification.

## 5. Discussion

Comparing the maximum accuracy of the binary classification, which is 96.4%, to the greatest accuracy of the multiclass classification, which is 93.4%, the multiclass classification has 3.21% less accuracy. This is due to the fact that multiclass classification is more difficult than binary classification. This result is consistent with the model described in Iqbal et al. (70) to distinguish between TB and non-TB patients based on a CXR. This study employed the same model to classify the binary class with an accuracy of 99.17%, while the multiclass classification model provided an accuracy of 95.10%, which was approximately 4.27% higher than the multiclass classification model. Since a multiclass classifier typically gives more weight to the largest classes, its global performance may not be indicative of how well it classifies a subset of the data (e.g., it may have 90% accuracy simply because class B is 90% of the data, but this does not prove anything about another class). The success of a binary classifier, on the other hand, can only ever be measured in relation to that class.

Table 4 shows that when data augmentation was used, the solution quality (accuracy) was greater than when data augmentation was not implemented. The variance was 8.1% (the classification with augmentation had higher accuracy). This conclusion is consistent with those obtained in Monshi et al. (71) and Barshooi et al. (72). To train our deep learning model, we needed more data, and image augmentation techniques provided this. We did not have to go out of our way to manually collect these new images because they were generated using the existing collection. When using augmentation techniques, more features can be gleaned from the primary dataset. Sometimes, image augmentation is used to prevent overfitting a model due to a small dataset. A deep learning model cannot learn so many patterns from a limited dataset. As a result, the model’s accuracy will suffer when applied to the classification of data not included in the training set. For this reason, image augmentation is commonly used to improve classification accuracy.

According to the findings of our study, lung segmentation improved the accuracy by around 27% compared to models that do not employ lung segmentation. This outcome is supported Karki et al. (15) and Tulo et al. (16). In these studies, an effective lung segmentation was established and incorporated into the model, with the results greatly outperforming those of prior studies. In our research, we partitioned the lungs using the U-Net model (31). As shown in Moor et al. (31), EfficientNetB7 was utilized in place of EfficientNetB4. The computational results of Moor et al. (31) also demonstrated that modified U-Net lung segmentation was successfully implemented and significantly improved the accuracy. Segmentation was a crucial step in the image recognition process, as it allowed us to isolate the features of interest in preparation for subsequent analyzes (description, recognition, etc.). Segmentation is frequently used for pixel classification in images. Segmentation techniques allowed us to isolate the object of interest in an image by erasing its surrounding context. Because of this, the accuracy of the categorization was greatly improved when lung segmentation was performed.

Adjusting the learning rate is another method to improve classification precision. Using CLR improved the classification accuracy by 5.3%. This result agreed with Smith (49). CLR was shown to be superior to the standard implementation of the learning rate. Due to the fact that saddle points, rather than local minima, are the primary challenge in optimizing deep neural networks, CLR stands out among the other types of learning rates. The learning process can be slowed by the insufficient slopes at saddle points. On the other hand, if the rate of learning is accelerated, saddle point plateaus can be quickly overcome. Our results showed that the new fusion technique, called HyVaN-AMIS, was superior to the standard UWA model. Using this method, the UMA’s solution quality could be boosted by 4.4%. It is assumed that various CNNs will be better fits for certain classes in the classification model; hence, HyVaN-AMIS assigns different weights to each CNN. According to Tiberi et al. (73), we can draw this conclusion. This study’s primary objective was to devise efficient fusion mechanisms for combining data from many intelligence boxes. The hybrid version of AMIS and VaNSAS was the most effective at finding the optimal solution without becoming trapped in the local optima because their structures were flexible enough to allow them to escape from the local optima (55, 56).

Deep ensemble learning models combine the benefits of both deep learning and ensemble learning, resulting in a model with superior performance (23). Depending on how it integrates the predictions of sub-models, ensemble learning can take several forms. Unweight average (UWA), or the averaging ensemble, aggregates the predictions from numerous trained models, although this method has the drawback that each sub-model contributes the same amount to the ensemble prediction, regardless of how well that sub-model performed individually. A weighted average ensemble is an approach for decision fusion in which the contribution of each sub-model is weighted based on the expected performance of the model on a portion of the test dataset. This ensures that sub-models with better performances contribute more, while sub-models with poorer performances contribute less. Any deep learning technique is offered to replace the linear weighted sum model used to integrate the predictions of the sub-models (74). This method is known as stacked generalization and employs the idea of the multilayer perceptron (MLP) (74).

In this stacking strategy, an algorithm takes the outputs of sub-models as inputs and attempts to learn how to optimally combine the input predictions to produce a more accurate output prediction. Three CNN-based models were utilized as sub-models, and a dense neural network was employed as a meta-learner that received input from all three sub-models and attempted to combine their predictions to outperform them separately (75). The stacked ensemble model suggested by Xie et al. (51) was constructed by stacking three distinct convolutional neural network (CNN) models. Electrocardiogram (ECG) signals were employed as inputs for training and evaluating the model. The outcome indicated that the suggested model can predict stroke with 99.7% accuracy. The F1 score, precision, and recall were, respectively, 99.69, 99.67, and 99.71%. This study concluded that with their approach, ECG can provide highly effective assistance in the identification of stroke. Therefore, the categorization of medical images using MLP-based techniques, such as stacking generalization and stacked ensemble models, has been shown to be advantageous. Additionally, CXR can be utilized efficiently for the categorization of drug-resistant tuberculosis. Effectively substituting the HyVaN-AMIS strategy for decision fusion with the MLP-based method is possible.

Patients were assigned a TB type at the time of data entry based on the profile of a drug sensitivity test (DST). TB types were defined by the WHO 2021 criteria (76) as follows: DS-TB has no resistance to anti-TB drugs, mono-DR-TB has resistance to only one first-line anti-TB drug, poly-DR-TB has resistance to more than one first-line anti-TB drug (other than isoniazid and rifampicin), MDR-TB has resistance to at least isoniazid and rifampicin, pre-XDR-TB is MDR-TB with additional resistance to any fluoroquinolone, and XDR-TB is pre-XDR-TB that is also resistant to one additional group A anti-TB drug (bedaquiline or linezolid). Compared to the latest WHO schema of drug-resistant TB classification (76), rifampicin-resistant (RR) and isoniazid-resistant (Hr) TB were not assigned to any TB Portals dataset. Due to the lack of detailed DST profiles to reorganize these cases, the information on TB types provided by the dataset was used without alteration. In addition, as RR-TB can overlap with other types of drug-resistant TB, while Hr-TB can be regarded as a subtype of mono-DR-TB, the two groups were not intended to be part of this TB classifier implementation.

The regimen that is used to treat drug-resistant patients changes from time to time. The regimen may be different when used with different patients at different times. The recommended regimen needs to be updated regularly according to country announcements or WHO announcements regarding the most updated regimen. The effectiveness of the medicine depends on the health condition of the patient and the medicine itself, which must be suitable for the condition of the patient (73). The pharmacist needs to regularly check the recommended regimen and update the application. The doctor or medical staff could use information from the recommended regimen given by the application as a guideline for the treatment program, but the medical staff also needs to consider the patient‘s personal information, individual drug response, and condition in order to provide the right medicine to that patient.

Artificial intelligence (AI) is becoming a more powerful tool in healthcare, offering new techniques to detect and suggest the option treatment of a variety of ailments. The AI application, TB-DRD-CXR assists physicians in diagnosing tuberculosis drug resistant types and making therapy suggestions. These apps can deliver results in a relatively short period of time, making them a cost-effective choice for healthcare professionals. The speed and accuracy of the results are two of the key advantages of adopting AI mobile applications for disease classification. These programs can analyze vast amounts of data in real time, allowing doctors to identify and treat ailments more rapidly and effectively. Besides speed and accuracy, AI mobile applications can also give clinicians with particular information and treatment recommendations. This can assist clinicians in making more informed treatment decisions and enhance patient outcomes. Another key benefit of adopting AI mobile applications for disease classification is their low cost. The TB-DRD-CXR can deliver accurate results without the need for costly equipment or considerable training. As a result, they are a viable option for healthcare practitioners, particularly those working in resource-constrained environments. Furthermore, by enhancing diagnostic accuracy and treatment suggestions, these apps have the potential to save healthcare costs by reducing unnecessary tests and procedures.

Trustworthiness is an important aspect in the successful adoption of TB-DRD-CXR in healthcare. To ensure widespread adoption, patients and healthcare professionals must have faith in the accuracy and dependability of these applications. Developers must guarantee that these applications are transparent, safe, and conform to privacy and ethical norms in order to build confidence. Finally, TB-DRD-CXR is a significant tool for supporting physicians in diagnosing drug resistance and offering precise treatment recommendations. Because of their speed, precision, and cost-effectiveness, they are an appealing choice for healthcare practitioners, particularly those working in resource-constrained situations. Therefore, it is critical to guarantee that TB-DRD-CXR are successfully integrated into clinical practice.

## 6. Conclusion and outlook

For this study, the TB-DRD-CXR web application was created. It is used to categorize TB patients into subgroups according to their level of drug resistance. The web app uses ensemble deep learning to categorize TB strains into five subtypes: DS-TB, DR-TB, MDR-TB, pre-XDR-TB, and XDR-TB. The ensemble deep learning now includes image segmentation, data augmentation, and several learning rate strategies in addition to an effective novel fusion technique. The suggested model was compared to both state-of-the-art approaches and to the standard homogeneous CNN architectures using different types of CNN architectures that are found in the literature.

The effectiveness of the model embedded in TB-DRD-CXR was subjected to various types of tests. First, the computational results indicated that the proposed model improved the accuracy by 33.9, 12.48, 4, and 12.2% over previous models for the binary classification of DR_TB, DS-TB, MDR-TB, and XDR-TB. In a second experiment, the multiclass classification model was tested. Sixteen combinations of parameters were tested, which were (1) using image segmentation vs. not using image segmentation, (2) using image augmentation vs. not using image augmentation, (3) using ADADELTA vs. CLR for learning rate optimization, and (4) using UWA vs. HyVaN-AMIS as the decision fusion strategy. The computational result showed that the model that used (1) image segmentation; (2) image augmentation; (3) CLR, for learning rate optimization; and (4) HyVaN-AMIS, as the fusion strategy, was the best model for classifying multiclass drug-resistant TB. The model provided a 21.86% better solution, on average, compared to other models, with maximum and minimum percentages of 1.9 and 43.5 percent, respectively. The use of image segmentation and data augmentation increased the solution quality by 25.8 and 8.1%, respectively. Using CLR increased the accuracy of the drug resistance classification by 4.2%. The new decision fusion strategy, which was HyVaN-AMIS, was presented in this paper, and it could increase the solution quality of the model by 2.3%.

In the third experiment, the proposed model was tested and compared with homogeneous ensemble deep learning using different CNN architectures. The computational result showed that the proposed model provided a superior solution for the multiclass classification model compared to DenseNet20 (69), NASNetMobile (61), EfficientNetB7 (59), EfficientNetV2B3 (77), EfficientNetV2M (77), and ConvNeXtSmal (53), with improvements of 28.8, 93.4, 2.99, 48.0, 4.4, and 7.6%, respectively.

The online application was created for usage by 33 medical personnel in Thailand’s northeastern region. The application’s performance metric was comprised of two or three KPIs. First, the accuracy of the web application was determined by randomly selecting 760 images from the Portal dataset; it was discovered that its accuracy in classifying drug-resistant organisms was 96.1%, with an error rate of 3.86%. Eventually, we tested how the users felt while using the application. We gave 10 tasks to users and determined that the average execution time for all tasks across all users was 7.57 min. When evaluating the web application, the time-based efficiency (ET) produced a goal/min value of 4.16 and an overall relative efficiency (ORE) of 100 percent. The final evaluation of the provided application displayed the SUS score that users assigned to the application. Ten negative and positive questions were provided to users, and the SUS score of the program was found to be 96.7 percent, which was relatively high. Therefore, it can be concluded that TB-DRD-CXR is an application that users will like using and will likely recommend to others. The best strategy to interpret the result of the fast image analysis is to report the drug resistance type with the highest probability throughout the whole live camera session.

When we utilized TB-DRD-CXR in the fast image analysis model, the results were relatively unstable. This is a shortcoming of this study. In order to achieve the most effective classification results, it is necessary to conduct in-depth research on how to categorize rapid picture analysis. In addition, research related to various CNN architectures should be integrated into the ensemble deep learning to achieve the greatest model performance. For instance, in order to save computational time, should we merge CNNs with sizes that are not significantly different, or should we ensemble architectures with diverse sizes? This field is still available for further research.

One of the drawbacks of this study is the fact that the Portal dataset does not include all types of drug resistance documented in the primary literature in medical journals. Therefore, in the future, the proposed model should be retrained with new data in addition to the data provided in the Portal dataset so that the classification result corresponds to the DR-TB types that are most common in the real world.

The second limitation relates to the drug-resistant TB protocol. Since the emergence of rapid molecular diagnostic tests and new and repurposed drugs, the treatment and management of drug-resistant tuberculosis (DR-TB) have developed significantly. Recent updates to the WHO’s treatment guidelines for MDR-TB are evidence-based. An administrator must follow the WHO or another health organization’s treatment regimen guidelines and regularly update the recommended regimen to ensure that it is the most recent regimen available when the DR-TB treatment regimen has changed. If the treatment regimen is not up to date, our proposed software will not be an auxiliary aid for physicians but rather a source of distraction.

As a potential extension of the current TB-DRD-CXR application, it would be beneficial to explore the possibility of implementing an AI system that can automatically detect drug-resistant types in photo galleries and provide the results in a more user-friendly manner. While the current application requires the user to browse and select the relevant image, an automated system that can recognize drug-resistant types directly from the phone’s photo album could significantly improve the efficiency and usability of the application. To achieve this goal, several steps can be taken, including providing camera capabilities in the user mobile app, integrating image recognition capabilities using machine learning or computer vision techniques, and activating image recognition when taking a photo. Such an extension would further enhance the transparency and explainability of the AI system, promoting its practical application in the field of TB classification.

In addition, as the datasets used in this study were labeled and their classifications were already known, it would be interesting to explore the potential of unsupervised learning techniques to further analyze the data. Unsupervised learning has been widely used in various domains, including computer vision and natural language processing, and can help discover hidden patterns and relationships in data without relying on labeled output. Applying unsupervised learning methods, such as clustering, to the labeled datasets used in this study could potentially reveal new insights into drug-resistant types and improve the accuracy of classification results. Specifically, exploring clustering algorithms, such as k-means, hierarchical clustering, and DBSCAN, could help group data points or objects based on their similarities or distances in a feature space, which could lead to the discovery of new drug-resistant types not previously identified by the label-based classification. Investigating these possibilities could be an exciting direction for future research in the field of TB classification and AI.

In computer vision and image processing, image augmentation and segmentation are two crucial techniques. Augmentation creates new training images by applying various transformations, while segmentation divides an image into multiple regions corresponding to objects or backgrounds. Combining these techniques can significantly improve the accuracy and robustness of image classification models. Segmentation identifies relevant image regions for classification, while augmentation improves generalization to input data variations. Consequently, models developed using effective augmentation and segmentation techniques produce superior results on real-world images. Such models are highly valuable in fields like medical imaging, autonomous vehicles, and surveillance. Therefore, exploring the development of models that leverage these techniques represents a promising research direction for enhancing image classification quality.

## Data availability statement

Publicly available datasets were analyzed in this study. The data presented in the study are deposited in TB Portal, accession via https://tbportals.niaid.nih.gov/. For TB-DRD-CXR, the link for code data is available at: https://github.com/aiosmartlab/TDRC.

## Ethics statement

Ethical review and approval was not required for the study on human participants in accordance with the local legislation and institutional requirements. Written informed consent from the [patients/participants or patients/participants legal guardian/next of kin] was not required to participate in this study in accordance with the national legislation and the institutional requirements.

## Author contributions

KS, RP, and NN contributed to conception and design of the study. SG, PE, and CK organized the database. CP, TP, RP, and SG performed the statistical analysis. RP and TP wrote the first draft of the manuscript. KS, TS, SK, NW, SJ, and NN wrote sections of the manuscript. All authors contributed to the article and approved the submitted version.

## Funding

This research was supported by the Research and Graduate Studies KhonKaen University, Thailand.

## Conflict of interest

The authors declare that the research was conducted in the absence of any commercial or financial relationships that could be construed as a potential conflict of interest.

## Publisher’s note

All claims expressed in this article are solely those of the authors and do not necessarily represent those of their affiliated organizations, or those of the publisher, the editors and the reviewers. Any product that may be evaluated in this article, or claim that may be made by its manufacturer, is not guaranteed or endorsed by the publisher.
